# Taurocholic acid inhibits the response to interferon-α therapy in patients with HBeAg-positive chronic hepatitis B by impairing CD8^+^ T and NK cell function

**DOI:** 10.1038/s41423-020-00601-8

**Published:** 2021-01-11

**Authors:** Zhen Xun, Jinpiao Lin, Qingqing Yu, Can Liu, Jinlan Huang, Hongyan Shang, Jianhui Guo, Yuchen Ye, Wennan Wu, Yongbin Zeng, Songhang Wu, Siyi Xu, Tianbin Chen, Jing Chen, Qishui Ou

**Affiliations:** 1 Department of Laboratory Medicine, Gene Diagnosis Research Center, The First Affiliated Hospital, Fujian Medical University, Fuzhou, China; 2 Fujian Key Laboratory of Laboratory Medicine, The First Affiliated Hospital, Fujian Medical University, Fuzhou, China; 3 First Clinical College, Fujian Medical University, Fuzhou, China; 4 Center of Liver Diseases, The First Affiliated Hospital, Fujian Medical University, Fuzhou, China

**Keywords:** Bile acids, Pegylated interferon, Hepatitis B virus, Immune cells, Mechanism, Hepatitis B, CD8-positive T cells, NK cells

## Abstract

Pegylated interferon-alpha (PegIFNα) therapy has limited effectiveness in hepatitis B e-antigen (HBeAg)-positive chronic hepatitis B (CHB) patients. However, the mechanism underlying this failure is poorly understood. We aimed to investigate the influence of bile acids (BAs), especially taurocholic acid (TCA), on the response to PegIFNα therapy in CHB patients. Here, we used mass spectrometry to determine serum BA profiles in 110 patients with chronic HBV infection and 20 healthy controls (HCs). We found that serum BAs, especially TCA, were significantly elevated in HBeAg-positive CHB patients compared with those in HCs and patients in other phases of chronic HBV infection. Moreover, serum BAs, particularly TCA, inhibited the response to PegIFNα therapy in HBeAg-positive CHB patients. Mechanistically, the expression levels of IFN-γ, TNF-α, granzyme B, and perforin were measured using flow cytometry to assess the effector functions of immune cells in patients with low or high BA levels. We found that BAs reduced the number and proportion and impaired the effector functions of CD3^+^CD8^+^ T cells and natural killer (NK) cells in HBeAg-positive CHB patients. TCA in particular reduced the frequency and impaired the effector functions of CD3^+^CD8^+^ T and NK cells in vitro and in vivo and inhibited the immunoregulatory activity of IFN-α in vitro. Thus, our results show that BAs, especially TCA, inhibit the response to PegIFNα therapy by impairing the effector functions of CD3^+^CD8^+^ T and NK cells in HBeAg-positive CHB patients. Our findings suggest that targeting TCA could be a promising approach for restoring IFN-α responsiveness during CHB treatment.

## Introduction

More than 240 million individuals worldwide are chronically infected with hepatitis B virus (HBV).^1^ If untreated, 15–40% of patients with chronic HBV infection progress to the onset of cirrhosis, which may lead to liver failure and liver cancer.^2^ Currently, interferons and nucleos(t)ide analogs are widely used to treat HBV.^3^ Among them, pegylated interferon-alpha (PegIFNα) provides the highest off-treatment sustained response (SR) rate and a relatively low risk of posttreatment relapse after a 48-week course of treatment in chronic hepatitis B (CHB) patients.^4^ Nevertheless, in hepatitis B e-antigen (HBeAg)-positive CHB patients who have received 48 weeks of PegIFNα therapy, a 24-week follow-up revealed SR rates of only 20–30%, and the rates of hepatitis B surface antigen (HBsAg) loss following 48 weeks of treatment were 3–7%.^4^ The underlying reason for this treatment resistance in CHB patients is still not well understood.

In chronic HBV infection, both innate and adaptive immune responses play pivotal roles in viral clearance.^5^ In innate and adaptive immune responses, natural killer (NK) cells and CD8^+^ T cells, respectively, are crucial in the defense against viral infections. NK and CD8^+^ T cells exert their antiviral effects against HBV infection through a range of mechanisms, including the release of cytolytic granules (granzyme and perforin) for the lysis of infected cells, induction of target cell apoptosis through crosslinking of cell-surface death receptors (FAS-FASL, TRAIL-TRAILR), and secretion of effector molecules (interferon-γ [IFN-γ] and tumor necrosis factor-α [TNF-α]).^6^ However, the activation of NK cells and subsequent production of IFN-γ and TNF-α are strongly hampered in CHB patients.^7^ In addition, HBV-specific CD8^+^ T cells are prone to apoptosis, and their ability to produce cytokines and proliferate is significantly reduced in CHB patients.^6^ Impaired CD8^+^ T-cell activity is a major contributor to persistent HBV infection.^8,9^ Taken together, previous evidence suggests that CHB results from an inadequate immune response, but the development of effective treatments requires a more detailed understanding of the mechanisms that impair NK and CD8^+^ T-cell responses during chronic HBV infection.

In recent years, growing evidence has pointed to the importance of the interregulation of cellular metabolism and the immune response.^10,11^ Bile acids (BAs) are cholesterol-derived amphipathic molecules that are produced in the liver and secreted into the duodenum. BAs facilitate digestion and absorption of lipids, regulate the metabolism of cholesterol, and promote bile secretion.^12^ BAs have been reported to regulate various cellular functions, such as the inhibition of NLRP3 inflammasome activation,^13^ enhancement of antiviral innate immunity,^14^ regulation of viral replication,^15,16^ and immunomodulatory effects of immune cells.^17–19^ Notably, BAs are signaling molecules that activate nuclear receptors to promote HBV gene expression, inhibit IFN-α activity in cell and animal models,^20–22^ and suppress human peripheral blood mononuclear cell (PBMC) activity in vitro.^23,24^ These results suggest that BAs have immunosuppressive effects on immune cells. However, little is known about the specific effects of BAs on the antiviral functions of NK and CD8^+^ T cells in CHB patients or whether BAs play a role in IFN-α therapy during CHB treatment.

In this study, we analyzed serum BA profiles in 110 patients with chronic HBV infection to explore the long-term outcome following IFN-α therapy in CHB patients. We then analyzed the frequency and effector functions of CD8^+^ T and NK cells in 50 HBeAg-positive CHB patients to investigate the immunomodulatory effects of BAs on immune cells. Finally, we focused on the role of a particular BA, taurocholic acid (TCA), and examined whether TCA inhibits IFN-α immunological activity by impairing the effector functions of CD8^+^ T and NK cells in vitro and in vivo. Taken together, our results provide a plausible immunological explanation as to why a majority of CHB patients have a poor therapeutic response to PegIFNα. Our findings may contribute to the development of novel therapeutic approaches to clear viral infections.

## Materials and methods

### Patients and healthy participants

A total of 130 participants were recruited at the First Affiliated Hospital of Fujian Medical University, including 110 patients with chronic HBV infection and 20 healthy controls (HCs) (Fig. S1). Based on the natural history of the chronic HBV infection, patients were classified according to four phases:^4^ HBeAg-positive chronic HBV infection, HBeAg-positive CHB, HBeAg-negative chronic HBV infection, and HBeAg-negative CHB. A summary of the patient characteristics is presented in Table S1.

Among the HBeAg-positive CHB patients, we enrolled 37 patients to be treated with PegIFNα for 48 weeks (Table S2). Following treatment, patients were assessed during a 24-week posttreatment observation period. Based on the curative effects in patients at the 24-week posttreatment (week 72) follow-up, HBeAg-positive CHB patients were divided into the SR (*n* = 18) and nonresponse (NR; *n* = 19) groups. An SR was defined according to previous studies^25,26^ as HBeAg < 1 S/CO, HBV DNA < 500 IU/ml, and ALT < 40 U/L. Patients were categorized as having a NR if they did not meet all of the above criteria.

This study was conducted in compliance with the 1975 Declaration of Helsinki and was approved by the Ethics Committee of the First Affiliated Hospital of Fujian Medical University (Approval No. MRCTA, ECFAH of FMU [2015]027). Written informed consent was obtained from the study participants.

### Serum BA profiling

The serum BA composition was measured at the Shanghai Metabolome Institute-Wuhan (Wuhan, China) using an ultrahigh-performance liquid chromatography/electrospray ionization tandem mass spectrometry system (1290-6470, Agilent Technologies, Inc. Santa Clara, CA, USA). The detailed experimental procedures are described in the Supplementary Materials and Methods.

### Establishment of a recombinant adeno-associated virus type 8 (rAAV8)-mediated HBV replication mouse model

rAAV8 carrying the 1.3-mer wild-type HBV genome (rAAV8-1.3HBV) was purchased from the FivePlus Molecular Medicine Institute (Beijing, China) and was used to establish an immunocompetent mouse model for chronic HBV infection.^27^ A total of 5 × 10^10^ viral genomes/200 μl virus were injected into the tail vein of each C57BL/6 mouse. The mice were bled every other week to monitor the HBsAg, HBeAg, and HBV DNA levels. After 6 weeks, mice were fed by oral gavage for 2 weeks with either 100-mg/kg TCA daily or a control diet. Following this, the mice were sacrificed.

### Detection of the expression of intracellular and cell-surface molecules by flow cytometry

For phenotypic analysis, human and mouse PBMCs were prepared and stained with monoclonal antibodies. For intracellular cytokine detection, after surface antigen staining, cells were fixed, permeabilized, and stained with anti-IFN-γ, anti-TNF-α, anti-perforin, and anti-granzyme B antibodies. The stained cells were analyzed using a Navios flow cytometer (Beckman), and the data were analyzed with FlowJo software. The detailed experimental procedures are described in the Supplementary Materials and Methods.

### In vitro cell culture and stimulation

Freshly isolated human PBMCs were cultured in 96-well plates at 37 °C in a 5% CO_2_ incubator. Cells were incubated in medium alone or with IFN-α (1000 U/ml; Beyotime Biotechnology) or IFN-α (1000 U/ml) plus TCA (100 μM; Sigma-Aldrich) for 24 h. Subsequently, cells were stimulated with phorbol 12-myristate 13-acetate (PMA) and ionomycin for 5 h, and then detection of the intracellular staining of IFN-γ, TNF-α, granzyme B, and perforin was performed by gating for NK and CD8^+^ T cells by flow cytometry.

### Statistical analysis

Data are expressed as the mean ± SD unless indicated otherwise. Data were analyzed with Prism 5 (GraphPad) software using the Mann–Whitney *U* test or unpaired *t* test when comparing two independent groups, the paired *t* test when comparing paired variables, and the *χ*^2^ test to examine differences in categorical variables. *P* < 0.05 was considered statistically significant.

## Results

### Serum TCA is significantly elevated in patients with HBeAg-positive CHB

**Fig. 1:**
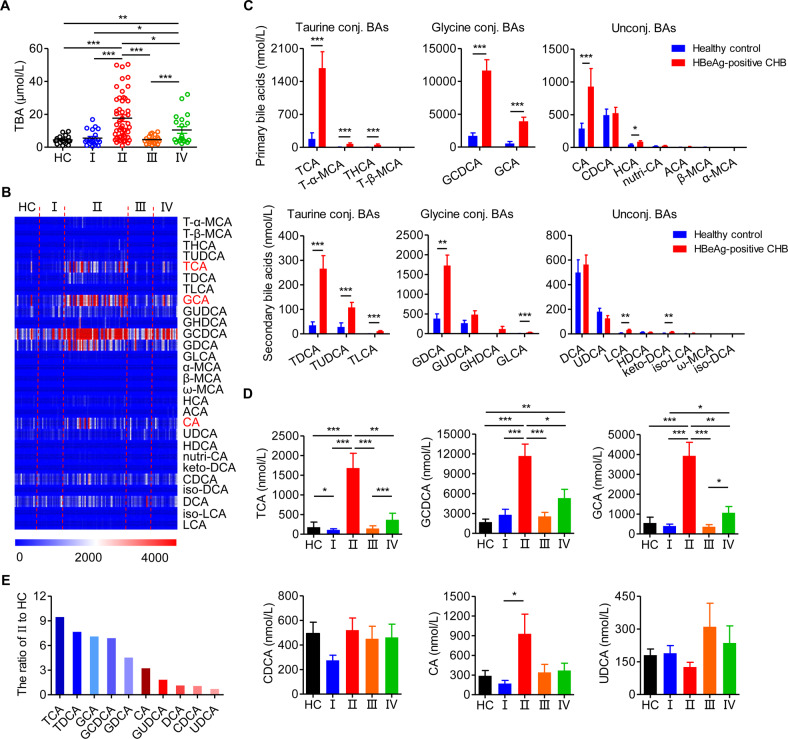
Serum taurocholic acid is significantly elevated in patients with HBeAg-positive chronic hepatitis B. **A** The level of total bile acids (TBAs) across the natural history of chronic HBV infection. **B** Heatmap of serum BA profiles in different disease phases. **C** The level of BAs in HCs and HBeAg-positive CHB patients. **D** The level of BAs in different disease phases. **E** The level of BAs in HBeAg-positive CHB (II) patients relative to that in healthy controls (HCs). HC, healthy control (*n* = 20); I, HBeAg-positive chronic HBV infection (*n* = 20); II, HBeAg-positive chronic hepatitis B (CHB) (*n* = 50); III, HBeAg-negative chronic HBV infection (*n* = 20); IV, HBeAg-negative chronic hepatitis B (*n* = 20). Data are presented as the mean ± SEM. Mann–Whitney *U* test. *p < 0.05, **p < 0.01, ***p < 0.001

Recent studies have demonstrated the dysregulation of BAs in various liver diseases, such as alcoholic hepatitis,^28^ nonalcoholic fatty liver disease,^29^ cirrhosis^30^ and HBV-related hepatocellular carcinoma,^31^ but the serum BA profiles associated with chronic HBV infection have not been previously reported. We used mass spectrometry to analyze the serum BA levels and BA composition in patients in different phases of chronic HBV infection who were naïve to treatment and HCs. The results of the BA measurements are depicted in Figs. 1 and S2. The levels of total and conjugated (taurine- and glycine-conjugated) BAs were significantly increased in HBeAg-positive CHB patients compared with those in HCs and patients in other phases of chronic HBV infection (Figs. 1A and S2). In contrast, there was no significant difference in the absolute concentrations of unconjugated BAs among the five groups (Fig. S2D).

To further assess the characteristics of the composition of BAs specific to HBeAg-positive CHB patients, the serum BA profiles were determined (Fig. 1B). BA composition analysis revealed that in HBeAg-positive CHB patients compared to HCs, there were significantly higher levels of the primary BAs TCA, tauro-α-muricholic acid, taurohyocholic acid, glycochenodeoxycholic acid (GCDCA), glycocholic acid (GCA), cholic acid (CA) and hyocholic acid and the secondary BAs taurodeoxycholic acid (TDCA), tauroursodeoxycholic acid, taurolithocholic acid, glycodeoxycholic acid (GDCA), glycolithocholic acid (GLCA), lithocholic acid, and ketodeoxycholic acid (Fig. 1C). Among these, only TCA, GCDCA, and GCA were significantly elevated in HBeAg-positive CHB patients compared with HCs and patients in other phases of chronic HBV infection (Fig. 1D). In particular, among the BAs, TCA levels most clearly differentiated HBeAg-positive CHB patients and HCs (Fig. 1E). Notably, there was no significant difference among the five groups in the absolute concentrations of BAs that have been the focus of previous research, including chenodeoxycholic acid (CDCA), CA, and ursodeoxycholic acid (UDCA) (Fig. 1D). Taken together, these results suggest that serum BAs, especially TCA, are significantly elevated in patients with HBeAg-positive CHB compared with those in HCs and patients in other phases of chronic HBV infection.

### TCA inhibits the response to interferon-α therapy in patients with HBeAg-positive CHB

**Fig. 2:**
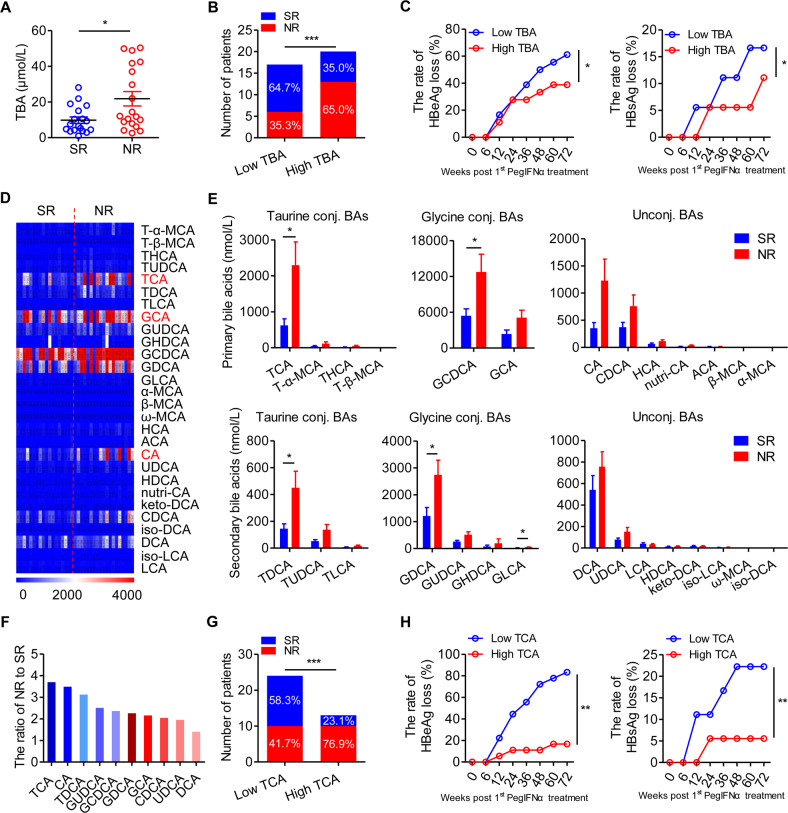
Taurocholic acid inhibits the response to interferon-α therapy in patients with HBeAg-positive chronic hepatitis B. **A** The level of serum total bile acid (TBA) in the sustained response (SR; *n* = 18) and nonresponse (NR; *n* = 19) groups of HBeAg-positive chronic hepatitis B (CHB) patients receiving pegylated interferon-alpha (PegIFNα) therapy. Mann–Whitney *U* test. **B** The number of SR and NR patients in the low TBA (*n* = 17) and high (*n* = 20) TBA groups. Chi-squared test. **C** The rate of HBeAg and HBsAg loss in the low TBA (*n* = 11) and high TBA (*n* = 7) groups in SR patients at the indicated time points after PegIFNα treatment. Two-way ANOVA. **D** Heatmap of serum BA profiles in SR and NR patients. **E** The level of BAs in the SR (*n* = 18) and NR (*n* = 19) groups. Mann–Whitney *U* test. **F** The level of BAs in the NR group relative to the SR group. **G** The number of SR and NR patients in the low taurocholic acid (TCA) group (*n* = 24) and high TCA group (*n* = 13). Chi-squared test. **H** The rate of HBeAg and HBsAg loss in the low TCA (*n* = 15) and high TCA (*n* = 3) groups in SR patients at the indicated time points after PegIFNα treatment. Two-way ANOVA. Data are presented as the mean ± SEM. *p < 0.05, **p < 0.01, ***p < 0.001

According to the guidelines,^4^ HBeAg-positive CHB patients should be treated. To identify whether BAs are associated with the anti-HBV treatment response, we compared serum BA levels in HBeAg-positive SR and NR patients who had received PegIFNα treatment and found that the NR group exhibited higher baseline total and taurine-conjugated BA levels (Figs. 2A and S3). In addition, among HBeAg-positive patients, we compared the low and high TBA patients and found that the SR rate of the high TBA group was significantly lower than that of the low TBA group (35.0% versus 64.7%, Fig. 2B). Furthermore, the rate of HBeAg and HBsAg loss in the low TBA group was consistently higher than that in the high TBA group throughout the course of treatment (Fig. 2C).

We next compared the serum BA profiles of HBeAg-positive SR and NR patients (Fig. 2D). BA composition analysis revealed significantly elevated primary BA (TCA and GCDCA) and secondary BA levels (TDCA, GDCA, and GLCA) in NR patients compared to those in SR patients, but there were no significant intergroup differences in CDCA, CA, or UDCA levels (Fig. 2E). Among the BAs, TCA levels most clearly differentiated between NR and SR patients (Fig. 2F). In addition, the SR rate for HBeAg-positive patients in the high TCA group was significantly lower than that in the low TCA group (23.1% versus 58.3%, Fig. 2G). Furthermore, the rate of HBeAg and HBsAg loss in the low TCA group was consistently higher than that in the high TCA group throughout the course of treatment (Fig. 2H). These findings suggest that serum BAs, especially TCA, inhibit the PegIFNα therapeutic response in HBeAg-positive CHB patients.

### TCA impairs the effector functions of CD3^+^CD8^+^ T and NK cells from patients with HBeAg-positive CHB

**Fig. 3:**
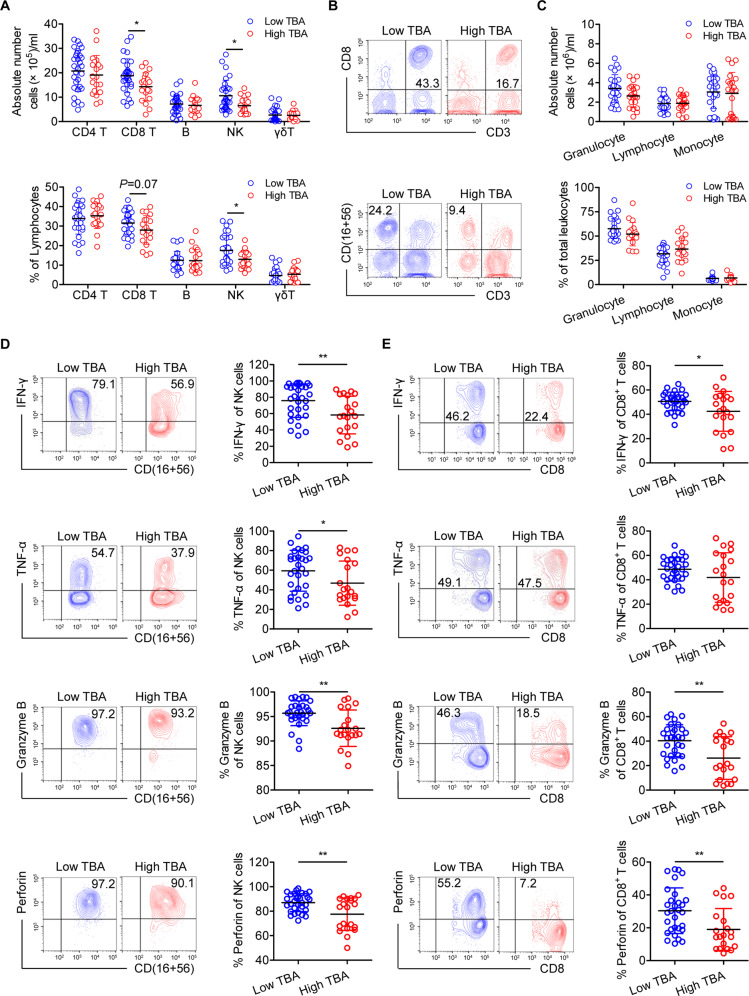
Bile acids impair the effector functions of CD3^+^CD8^+^ T and NK cells in HBeAg-positive chronic hepatitis B patients. **A** Quantification of CD3^+^CD4^+^ T cells, CD3^+^CD8^+^ T cells, B cells, NK cells, and γδ^+^ T cells in HBeAg-positive CHB patients with low (*n* = 30) or high (*n* = 20) TBA levels. The absolute number of immune cells was calculated by multiplying the total number of peripheral blood leukocytes by the percentage of positive cells. **B** Representative flow cytometry (FCM) profiles of CD3^+^CD8^+^ T and NK cells are shown for each group. **C** The number and proportion of granulocytes, lymphocytes, and monocytes within the total leukocyte population were determined by a hematology analyzer for HBeAg-positive CHB patients with low (*n*=30) or high (*n*=20) TBA levels. **D**, **E** Freshly isolated PBMCs from HBeAg-positive CHB patients with low (*n*=30) or high (*n*=20) TBA levels were stimulated with PMA and ionomycin for 5 h. Representative FCM profiles and quantification of the expression of the indicated molecules in NK cells (**D**) and CD3^+^CD8^+^ T cells (**E**). Unpaired *t* test. *p < 0.05, **p < 0.01, ***p < 0.001

Accumulating evidence suggests that in HBV treatment, IFN-α exerts its antiviral effects by enhancing the effector functions of NK and CD8^+^ T cells.^32^ Based on the poor PegIFNα treatment response we observed in CHB patients with high TBA levels, we hypothesized that BAs may impair the effector functions of immune cells. We compared the number of immune cell populations in peripheral blood in HBeAg-positive CHB patients in the low TBA and high TBA groups and found that the high TBA group had lower numbers and proportions of CD3^+^CD8^+^ T and NK cells (Fig. 3A, B), but there were no significant intergroup differences in the numbers or proportions of granulocytes, lymphocytes, or monocytes (Fig. 3C). To further assess the effector functions of CD3^+^CD8^+^ T and NK cells, we characterized the markers associated with degranulation and cytokine production. There were reduced expression levels of IFN-γ, TNF-α, granzyme B, and perforin in CD3^+^CD8^+^ T and NK cells from patients with high TBA levels compared with those in cells from patients with low TBA levels (Fig. 3D, E), indicating the reduced cytokine production and degranulation in CD3^+^CD8^+^ T and NK cells from patients with higher TBA levels. These findings demonstrate that BAs reduce the proportions and effector functions of CD3^+^CD8^+^ T and NK cells in HBeAg-positive CHB patients.

**Fig. 4:**
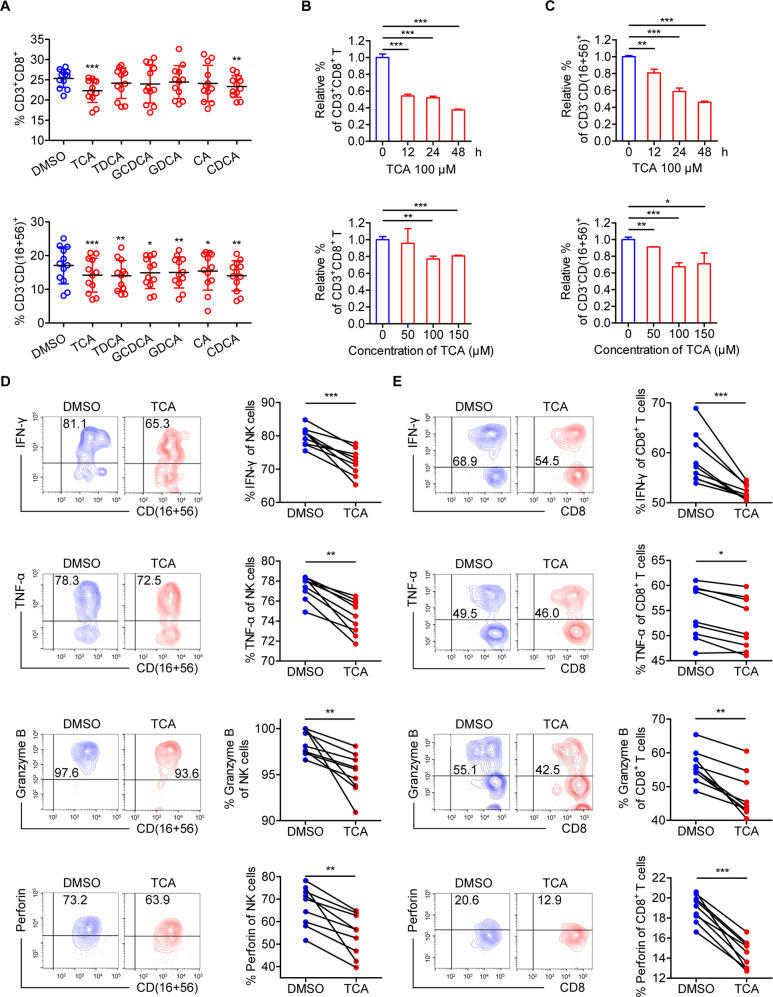
Taurocholic acid impairs the effector functions of CD3^+^CD8^+^ T and NK cells from patients with HBeAg-positive chronic hepatitis B in vitro. **A** Freshly isolated PBMCs from HBeAg-positive chronic hepatitis B (CHB) patients (*n* = 12) were stimulated with or without a variety of BAs (100 μM). Cultured cells were harvested after 12 h and analyzed. The proportions of CD3^+^CD8^+^ T cells and NK cells were analyzed by flow cytometry. Paired *t* test. **B**, **C** The relative proportions of CD3^+^CD8^+^ T cells (**B**) and NK cells (**C**) were analyzed by flow cytometry in freshly isolated PBMCs stimulated with 100-μM taurocholic acid (TCA) for the indicated times or stimulated with TCA at different concentrations for 24 h. Unpaired *t* test. **D**, **E **Freshly isolated PBMCs from CHB patients (*n* = 9) were stimulated with PMA and ionomycin for 5 h after stimulation with or without TCA (100 μM) for 24 h, and intracellular staining of IFN-γ, TNF-α, granzyme B, and perforin was determined using flow cytometry by gating on NK cells (**D**) and CD3^+^CD8^+^ T cells (**E**). Paired *t* test. *p < 0.05, **p < 0.01, ***p < 0.001

Next, we examined the effect of specific BAs on immune cells by stimulating freshly isolated PBMCs from HBeAg-positive CHB patients with several typical BAs, including TCA, TDCA, GCDCA, GDCA, CA, and CDCA. TCA had the greatest effect on the proportion of CD3^+^CD8^+^ T and NK cells (Fig. 4A), and TCA levels also showed the greatest variation between the different phases of chronic HBV infection (Fig. 1E) and between SR and NR patients (Fig. 2F). The effects of TCA on CD3^+^CD8^+^ T and NK cells were further assessed via treatment with different concentrations of TCA and incubation for different lengths of time of freshly isolated PBMCs from HBeAg-positive CHB patients. We observed a time-dependent decrease in the proportion of CD3^+^CD8^+^ T and NK cells following 100-μM TCA treatment (Fig. 4B, C). In addition, for PBMCs stimulated for 24 h, 100-μM TCA had the largest effect on the numbers of CD3^+^CD8^+^ T and NK cells (Fig. 4B, C). The decrease caused by TCA was not due to changes in cell proliferation or apoptosis (Fig. S4). Moreover, TCA did not affect the proportions of CD3^+^CD8^+^ T and NK cells from HCs (Fig. S5). To further explore the effect of TCA on the effector functions of CD3^+^CD8^+^ T and NK cells, after freshly isolated PBMCs from HBeAg-positive CHB patients were stimulated with 100-μM TCA for 24 h, we performed intracellular staining of IFN-γ, TNF-α, granzyme B, and perforin. The results indicated that stimulating CD3^+^CD8^+^ T and NK cells with TCA led to lower levels of cytokines and cytotoxic granules compared to those in controls (Fig. 4D, E). These results demonstrate that TCA reduces the frequency and effector functions of CD3^+^CD8^+^ T and NK cells from HBeAg-positive CHB patients in vitro.

### TCA impairs the effector functions of CD3^+^CD8^+^ T and NK cells in vivo

**Fig. 5:**
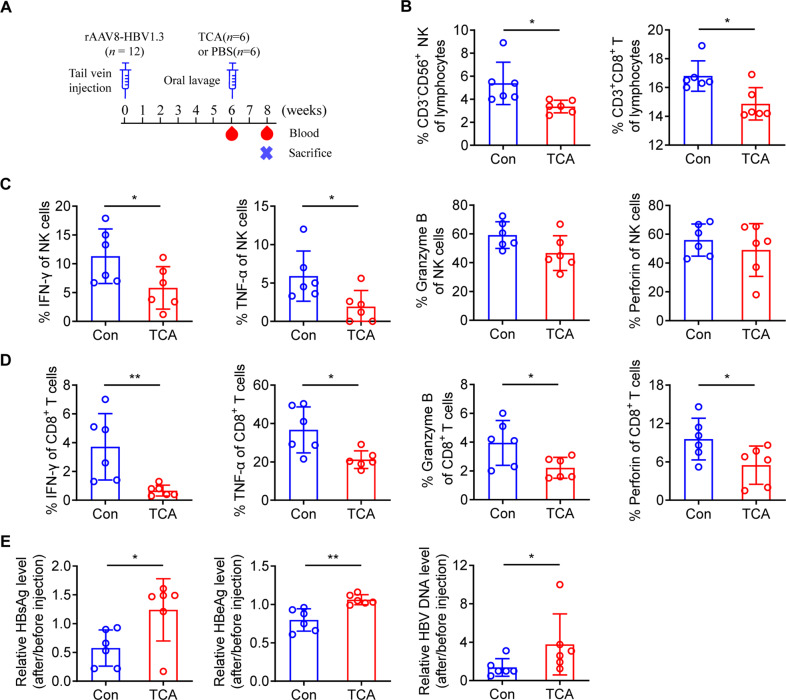
Taurocholic acid impairs the effector functions of CD3^+^CD8^+^ T and NK cells in vivo. **A** Schematic representation of HBV markers and immune cell detection. **B** After 2 weeks of taurocholic acid (TCA) administration (100 mg/kg/day), the proportions of NK and CD3^+^CD8^+^ T cells were analyzed by flow cytometry.** C**, **D** After 2 weeks of TCA administration, freshly isolated PBMCs from C57BL/6 mice were stimulated with PMA and ionomycin for 5 h, and then intracellular staining of IFN-γ, TNF-α, granzyme B, and perforin was determined using flow cytometry by gating on NK cells (**C**) and CD3^+^CD8^+^ T cells (**D**). **E** After 2 weeks of TCA administration, serum HBsAg, HBeAg, and HBV DNA levels were measured. Unpaired *t* test. *p < 0.05, **p < 0.01

To determine whether TCA suppresses the effector functions of CD3^+^CD8^+^ T and NK cells in vivo, we gavaged C57BL/6 mice with 100-mg/kg TCA daily or a control diet for 2 weeks after tail vein injection with rAAV8-1.3HBV for 6 weeks (Fig. 5A). The serum level of TCA was significantly elevated after gavage (Fig. S6). We found that treatment with TCA significantly reduced the percentage of NK and CD3^+^CD8^+^ T cells (Fig. 5B). In addition, CD8^+^ T and NK cells from C57BL/6 mice treated with TCA produced lower levels of cytokines and cytotoxic granules than those from mice given a control diet (Fig. 5C, D). Importantly, compared to those given the control diet, mice treated with TCA had higher serum HBsAg, HBeAg, and HBV DNA levels (Fig. 5E). These findings indicate that TCA promotes HBV replication by decreasing the percentage and impairing the effector functions of CD3^+^CD8^+^ T and NK cells in vivo.

### TCA inhibits the immunoregulatory activity of IFN-α in vitro

**Fig. 6:**
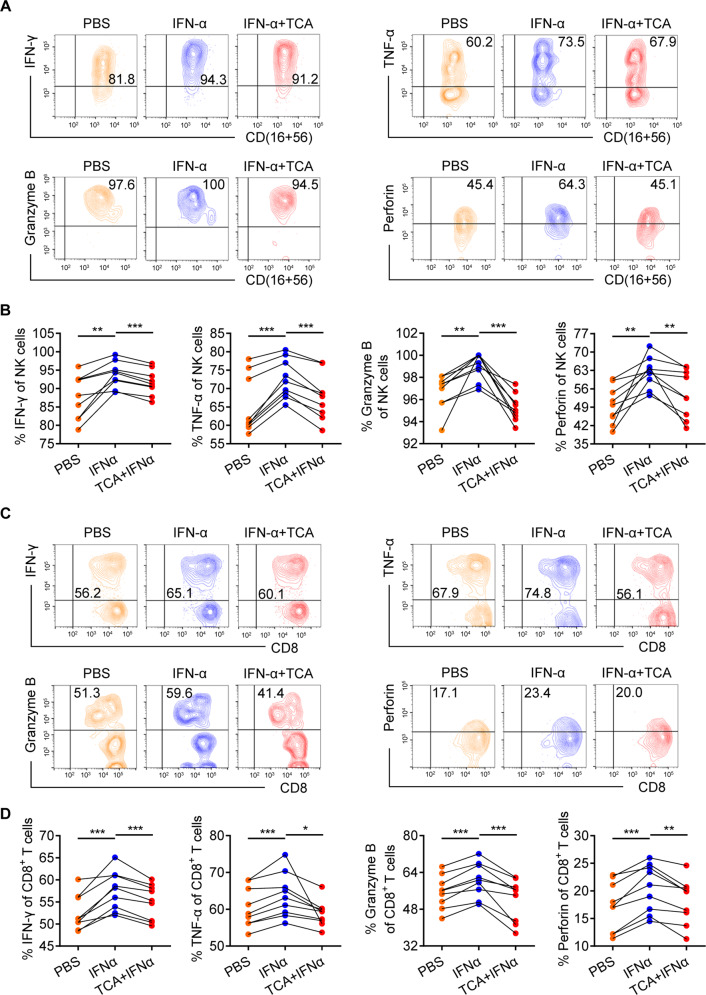
Taurocholic acid inhibits the immunomodulatory activity of IFN-α in vitro. **A**, **C **Representative density plots showing the analysis of IFN-γ, TNF-α, granzyme B, and perforin expression in gated NK cells (**A**) and CD3^+^CD8^+^ T cells (**C**). Data are representative of three independent experiments. **B**, **D** Freshly isolated PBMCs from HBeAg-positive chronic hepatitis B patients (*n* = 9) were stimulated with PMA and ionomycin for 5 h after stimulation with or without IFN-α (1000 U/ml) or TCA (100 μM) plus IFN-α for 24 h. Intracellular staining of IFN-γ, TNF-α, granzyme B, and perforin was assessed using flow cytometry by gating on NK cells (**B**) and CD3^+^CD8^+^ T cells (**D**). Data are pooled from three independent experiments. Paired *t* test. *p < 0.05, **p < 0.01, ***p < 0.001

Given that IFN-α is an important immunomodulator^33^ and that our results indicate that TCA suppresses both the response to IFN-α therapy in CHB patients and the effector functions of CD3^+^CD8^+^ T and NK cells in vitro and in vivo (Figs. 2–5), we postulated that TCA inhibits IFN-α function by inhibiting its immunoregulatory activity. To test this hypothesis, due to the lack of appropriate and convenient animal models of HBeAg-positive CHB,^34^ we first stimulated freshly isolated PBMCs from HBeAg-positive CHB patients with IFN-α or TCA plus IFN-α for 24 h. We then performed intracellular staining of IFN-γ, TNF-α, granzyme B, and perforin in CD3^+^CD8^+^ T and NK cells and found that CD3^+^CD8^+^ T and NK cells stimulated with IFN-α produced higher levels of cytokines and cytotoxic granules than control cells (Fig. 6), which was consistent with previous work.^35,36^ Moreover, CD3^+^CD8^+^ T and NK cells that were stimulated with TCA plus IFN-α produced lower cytokine and cytotoxic granule levels than those that were stimulated with IFN-α alone (Fig. 6). Overall, these findings indicate that TCA inhibits the immunomodulatory effects of IFN-α in vitro.

## Discussion

HBeAg-positive CHB patients have low SR rates to PegIFNα therapy, and the reasons for this treatment resistance are still not fully understood.^3^ Previous reports have shown that BAs can modulate the interferon signaling pathway in hepatoma cell lines.^37^ In addition, BAs can increase HBV gene expression and inhibit IFN-α activity in 1.2-mer HBV replicon-transfected HepG2 cells.^20^ However, few studies have investigated the role of BAs in CHB patients receiving PegIFNα therapy.

In this study, we measured the serum BA profiles during the natural progression of chronic HBV infection. Our results showed that serum BAs, especially TCA, are significantly elevated in HBeAg-positive CHB patients compared with those in HCs and patients in other phases of chronic HBV infection, which is consistent with previous findings.^38^ This effect may be due to impaired liver function in active chronic hepatitis.^4^ Both the innate and adaptive immune responses induced by HBV infection can lead to hepatocyte damage and inflammatory necrosis, which is accompanied by the release of BAs from liver cells into the systemic circulation.^39^ In addition, the increase in the levels of conjugated primary BAs (TCA, GCDCA, and GCA) in HBeAg-positive CHB patients can in part be explained by the presence of gut microbiota dysbiosis in HBV-induced chronic liver disease.^40^ Normally, conjugated CA and CDCA are deconjugated via bile salt hydrolases to form unconjugated CA and CDCA, and these primary BAs are metabolized by bacterial 7α-dehydroxylase to form secondary BAs (CA to DCA and CDCA to LCA) in the intestine.^12^ The main bacterial genera of the gut microbiota involved in BA metabolism include *Bacteroides*, *Clostridium*, *Lactobacillus*, *Bifidobacterium,* and *Listeria*, which are involved in BA deconjugation, and *Clostridium* and *Eubacterium*, which are involved in 7-dehydroxylation.^12^ However, *Clostridium*, *Bifidobacterium,* and *Eubacterium* abundance was reduced in CHB patients.^40^ Furthermore, we found that the absolute concentrations of unconjugated CDCA and CA did not differ among the phases of chronic HBV infection, indicating that unconjugated CDCA and CA play major roles under physiological conditions, while conjugated CDCA and CA play the main pathological role in the pathological state.

Current studies have shown that BAs can modulate the interferon signaling pathway.^37^ However, it is not well understood whether BAs modulate the therapeutic response to PegIFNα in CHB patients. Here, we demonstrated that HBeAg-positive CHB patients with high BA levels exhibit a poor therapeutic response to PegIFNα. High levels of TCA in particular predicted a poor response to PegIFNα. These findings suggest that TCA inhibits the response to IFN-α therapy in HBeAg-positive CHB patients. The mechanisms governing the metabolic regulation of the anti-HBV effect of IFN-α mainly focus on the JAK-STAT pathway.^37,41^

Current research suggests that IFN-α induces the production of antiviral proteins through the JAK-STAT pathway and can also inhibit virus replication through immune regulation.^33,42^ Here, we showed that compared to patients with low TBA levels, patients with high TBA levels have a reduced number and proportion of CD8^+^ T and NK cells, defective degranulation, and decreased IFN-γ and TNF-α production. These findings demonstrate that BAs reduce the frequency and impair the effector functions of CD8^+^ T and NK cells in HBeAg-positive CHB patients, and this was particularly the case for TCA both in vitro and in vivo. BAs have a consistent immunosuppressive effect on human PBMCs in vitro and in vivo.^23,43^ In particular, TCA suppresses the immunoregulatory activity of IFN-α in vitro, which may lead to the poor efficacy of IFN-α treatment. Similar to our results, BAs suppress the biological activity of IFN-α and NK cell activity.^24^ Although the mechanisms of how TCA specifically affects CD8^+^ T and NK cell function need to be further explored, we consider three mechanisms to be the main likely contributors. First, BAs may exert their effects by intercalating in cell membranes to modify membrane fluidity and permeability, particularly toward calcium ions, and by activating specific transduction pathways such as the protein kinase C signaling pathway.^44^ Second, BAs may act by inhibiting the interaction between cytokine receptors and their ligands on CD8^+^ T and NK cell membranes or by inhibiting intracellular pathways involved in CD8^+^ T and NK cell activation.^43^ Third, TCA may affect CD8^+^ T and NK cell function through the major BA-sensing receptor Takeda G protein coupled receptor-5 (TGR5). The activation of TGR5 by TCA may upregulate the expression of the inhibitory receptor programmed death 1 (PD1) on CD8^+^ T cells via suppression of the production of interleukin-12^12,45,46^ and downregulate the expression of the activating receptor NKG2D on NK cells via induction of the production of transforming growth factor β,^12,47^ leading to the functional impairment of CD8^+^ T and NK cell function. Our results also revealed that TCA upregulates the expression of PD1 on CD8^+^ T cells and downregulates the expression of NKG2D on NK cells in HBeAg-positive CHB patients (Fig. S7).

Currently, there are no targeted drugs that reduce the level of TCA. UDCA is clinically used to reduce total BA levels and can also reduce the TCA level. However, UDCA therapy may cause several side effects, such as gastrointestinal complaints (diarrhea, dyspepsia, abdominal pain, flatulence, nausea, and vomiting), pruritus, urticaria, and gallstone calcification in addition to reducing the number of activated T cells.^23,43,48^ Moreover, various BAs have different physiological functions.^12,49^ Reducing the level of total BAs can affect the important physiological functions of some BAs, such as the absorption of dietary fats, the regulation of lipid, glucose, and energy metabolism, and the immune response.^12,17,19^ In the present study, we found that among the BAs, TCA levels most clearly differentiated between HBeAg-positive patients with a SR to PegIFNα treatment and those who were nonresponsive. To a certain extent, targeting TCA alone can prevent several side effects caused by UDCA therapy and also preserve the physiological functions of other BAs. Therefore, drugs targeting TCA have potential clinical significance.

The current findings point to the need for specific follow-up research. First, randomized controlled trials are needed to determine whether treatments that reduce TCA levels can improve the effector functions of CD3^+^CD8^+^ T and NK cells and the therapeutic response to PegIFNα in HBeAg-positive CHB patients. Second, the mechanisms involved in the inhibitory effects of BAs on immune cells deserve further study.

**Fig. 7:**
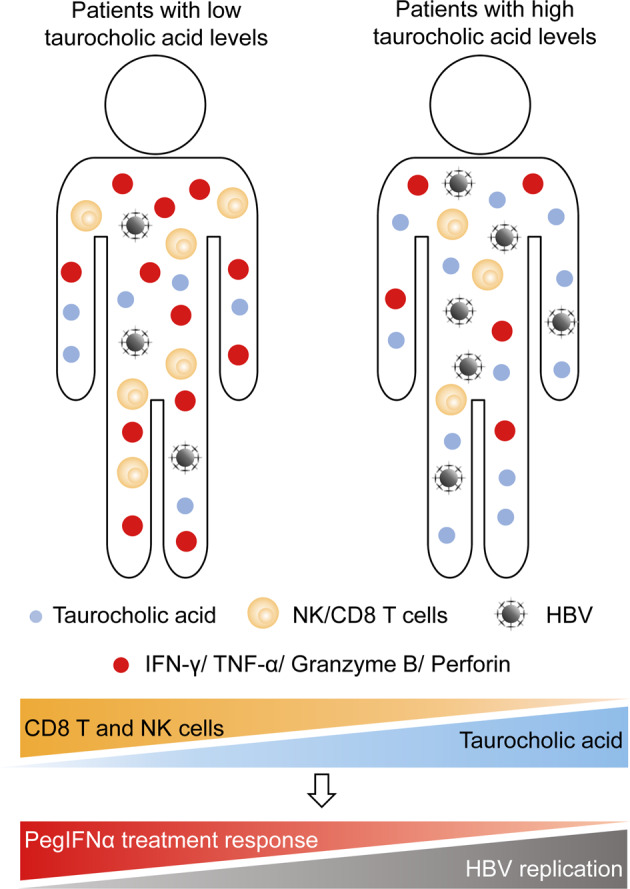
A model describing the mechanism regarding the inhibitory effect of taurocholic acid on the response to interferon-α therapy in patients with chronic hepatitis B. Compared with chronic hepatitis B (CHB) patients with low taurocholic acid (TCA) levels, CHB patients with high TCA levels had a lower number of CD3^+^CD8^+^ T and NK cells and lower levels of IFN-γ, TNF-α, granzyme B, and perforin secreted by CD3^+^CD8^+^ T and NK cells, resulting in the poor therapeutic response to pegylated interferon-alpha (PegIFNα) in CHB patients

In conclusion, the current study provides evidence of the nature and effect of variable serum BA profiles throughout the natural progression of chronic HBV infection, especially in HBeAg-positive CHB patients treated with PegIFNα. We found that in HBeAg-positive CHB patients, serum BAs, particularly TCA, inhibited the response to PegIFNα therapy by impairing the effector functions of CD3^+^CD8^+^ T and NK cells (Fig. 7). Thus, our results reveal a novel mechanism involved in the poor therapeutic response to PegIFNα and suggest that targeting TCA could be a promising approach for restoring the immunological functions of IFN-α during CHB treatment.

## Supplementary information

Supplementary Materials and Methods

Supplementary Table 1

Supplementary Table 2

Supplementary Figures 1-7
