# Supplementary material for: Taurocholic acid inhibits the response to interferon-α therapy in patients with HBeAg-positive chronic hepatitis B by impairing CD8+ T and NK cell function
Source: Cell Mol Immunol. 2021 Jan 11;18(2):461–71. doi: 10.1038/s41423-020-00601-8 (PMC8027018; doi:10.1038/s41423-020-00601-8)
Supplement: Supplementary file 1 — Supplementary Materials and Methods [file 41423_2020_601_MOESM1_ESM.doc]

**Supplementary Materials and Methods**

***Laboratory measurement of clinical indicators***

HBV DNA was detected using quantitative real-time PCR (Sansure Biotech Inc., Hunan, China) and a Roche Lightcycler 480 (Roche Corporation, Basel, Switzerland). HBsAg and HBeAg were quantified using an automated chemiluminescent microparticle immunology analyzer (Abbott I2000, Abbott Laboratories, Chicago, IL, USA). Alanine aminotransferase (ALT) and total bile acid (TBA) were quantified using an automatic biochemical analyzer ADVIA 2400 (Siemens, Munich, Germany).

***Serum bile acid profiling***

Fresh human serum was snap frozen in liquid nitrogen and then kept at -80 °C. Serum levels of total, conjugated and unconjugated as well as specific bile acids (BAs) were measured at the Shanghai Metabolome Institute-Wuhan (Wuhan, China) using an ultra-high-performance liquid chromatography/electrospray ionization tandem mass spectrometry (UPLC-ESI-MS/MS) system (1290-6470, Agilent Technologies, Inc. Santa Clara, CA, USA) as described previously [1]. In brief, samples (1 μl) were separated using a Kinetex Core-Shell 2.6 μm C18 column (100×2.1 mm, 2.6 μm, Phenomenex Inc. Torrance, CA) equipped with Kinetex 2.6 μm Minibore Security Guard Ultra Cartridges (Phenomenex Inc. Torrance, CA) at 45 °C. The mobile phases consisted of A (water with 0.005% HCOOH, v/v) and B (acetonitrile with 0.005% HCOOH, v/v). The stepwise elution gradient process was as follows: 1.23% B to 33% B for 2 min; 2.33% B to 34% B for 4 min; 3.34% B to 70% B for 5 min. The flow rate was 0.6 mL/min. Mass spectrometry (MS) detection of BAs was conducted in negative ion mode. Fragmentor and product ions for every BA were optimized through the direct infusion of available BA standards to improve detection sensitivity. Due to the low proportion of some bile acids in samples, we chose [M-H]^-^ as the product ion to promote sensitivity under multiple reaction monitoring (MRM) scan mode. Data acquisition and analysis were performed with Mass Hunter software (Agilent Technologies, Inc. Santa Clara, CA).

***Expression of intracellular and cell surface molecules by flow cytometry***

Human peripheral blood mononuclear cells (PBMCs) from chronic HBV infection and healthy donors were isolated by Ficoll-Hypaque (Solarbio, Beijing) density-gradient centrifugation, and mouse PBMCs were isolated by Percoll interface (Solarbio, Beijing) density-gradient centrifugation. After washing twice in PBS, PBMCs were resuspended in complete RPMI 1640 medium consisting of 10% FBS (both from Life Technologies), 100 U/ml penicillin, and 100 mg/ml streptomycin.

For phenotypic analysis, human and mouse PBMCs were prepared and stained with monoclonal antibodies (mAbs). Human PBMCs were stained with mAbs directed against CD3 (SK7), CD4 (RPA-T4), CD8 (SK1) and TCR gamma/delta (B1.1) (eBioscience, USA); CD19 (HIB19), PD1 (EH12.2H7) and NKG2D (1D11) from Biolegend; CD3CD(16+56) from Beckman Coulter, USA. Murine peripheral blood lymphocytes were stained with mAbs directed against CD3e (500A2) and NK1.1 (PK136) from eBioscience; CD8 (53-6.7) from Biolegend. The control cells were stained with corresponding isotype-matched control antibodies.

For intracellular cytokine detection, freshly isolated human and mouse PBMCs were cultured with RPMI 1640 supplemented with 10% FBS in the presence of Cell Stimulation Cocktail plus protein transport inhibitors (including PMA, ionomycin, monensin and Brefeldin A) (eBioscience). After culturing for 5 h at 37 ˚C and 5% CO_2_, cells were harvested and stained for surface antigens (Ags) using the anti-human CD3CD(16+56), anti-human CD8 (SK1), anti-mouse CD3e (500A2), anti-mouse CD8 (53-6.7) and anti-mouse NK1.1 (PK136) mAbs for 30 min at 4 ˚C in the dark. After fixation and permeabilization, cells were stained with anti-human IFN gamma (4S.B3), anti-human TNF alpha (MAb11), anti-mouse TNF alpha (MP6-XT22), anti-mouse IFN gamma (XMG1.2) (eBioscience); anti-human perforin (dG9), anti-human/mouse granzyme B (QA16A02), anti-mouse perforin (S16009A) (Biolegend) for 30 min at 4 ˚C in the dark, washed twice with permeabilization buffer, and analyzed by flow cytometry. Appropriate isotype Abs were used as controls for intracellular cytokine detection. Stained cells were analyzed using a Navios flow cytometer (Beckman), and data were analyzed with FlowJo software.

**Cell counting kit-8 (CCK-8) assay**

The cell viability was assessed by CCK8 (Beyotime Biotechnology, Shanghai, China) according to the manufacturer’s protocol. All of the experiments were performed in sixth. The cytotoxicity curves were plotted using the absorbance at each time point.

**Cell apoptosis analysis**

The apoptosis assay was done with the Annexin V-FITC/PI Apoptosis Detection Kit (Bioworld Technology, Nanjing, Jiangsu Province, China) according to the manufacturer’s instructions. To detect the effect of TCA on immune cell apoptosis, freshly isolated PBMCs from HBeAg-positive chronic hepatitis B (CHB) patients were stimulated with or without 100 μM TCA for 24 h, stained with a combination of annexin V and propidium iodide (PI), and analyzed by Navios flow cytometer (Beckman Coulter, USA).

**References**

[1] Lin H, An Y, Tang H, Wang Y. Alterations of Bile Acids and Gut Microbiota in Obesity Induced by High Fat Diet in Rat Model. J Agric Food Chem 2019;67:3624-3632.
