# Supplementary material for: Taurocholic acid inhibits the response to interferon-α therapy in patients with HBeAg-positive chronic hepatitis B by impairing CD8+ T and NK cell function
Source: Cell Mol Immunol. 2021 Jan 11;18(2):461–71. doi: 10.1038/s41423-020-00601-8 (PMC8027018; doi:10.1038/s41423-020-00601-8)
Supplement: Supplementary file 2 — Supplementary Table 1 [file 41423_2020_601_MOESM2_ESM.doc]

**Table S1. Baseline demographics and clinical characteristics of the study population**

| **Variables** | **Healthy**  **control** | **HBeAg-positive**  **chronic HBV infection** | **HBeAg-positive**  **chronic hepatitis B** | **HBeAg-negative**  **chronic HBV infection** | **HBeAg-negative**  **chronic hepatitis B** |
| --- | --- | --- | --- | --- | --- |
| **Number of participants** | 20 | 20 | 50 | 20 | 20 |
| **Clinical parameters** | | | | | |
| Gender, *n* Male/*n* Female | 10/10 | 11/9 | 32/18 | 10/10 | 14/6 |
| Age, years | 29±5 | 32±8 | 29±4 | 37±5 | 37±10 |
| **Laboratory parameters** | | | | | |
| Log_10_ HBV DNA, IU/ml | - | 7.6±0.4 | 7.4±0.8 | < 2.7 | 5.0±1.3 |
| Log_10_ HBsAg, IU/ml | - | 4.6±0.3 | 4.2±0.7 | 2.6±0.9 | 3.0±1.1 |
| Log_10_ HBeAg, S/CO | - | 3.1±0.2 | 3.0±0.3 | -0.4±0.1 | -0.5±0.1 |
| ALT, U/L | 16±6 | 27±11 | 282±205 | 30±8 | 130±49 |

Data are mean ± SD unless otherwise indicated. HBV, hepatitis B virus; HBeAg, hepatitis B e antigen; HBsAg, hepatitis B surface antigen; ALT, alanine aminotransferase.
