# Supplementary material for: Taurocholic acid inhibits the response to interferon-α therapy in patients with HBeAg-positive chronic hepatitis B by impairing CD8+ T and NK cell function
Source: Cell Mol Immunol. 2021 Jan 11;18(2):461–71. doi: 10.1038/s41423-020-00601-8 (PMC8027018; doi:10.1038/s41423-020-00601-8)
Supplement: Supplementary file 3 — Supplementary Table 2 [file 41423_2020_601_MOESM3_ESM.doc]

**Table S2. Baseline demographics and clinical characteristics of HBeAg-positive CHB patients treated with PegIFNα**

Data are mean ± SD unless otherwise indicated. HBeAg, hepatitis B e antigen; CHB, chronic hepatitis B; PegIFNα, pegylated interferon-alfa; SR, sustained response; NR, non-response; HBsAg, hepatitis B surface antigen; ALT, alanine aminotransferase.

| **Variables** | **SR (*n*=18)** | **NR (*n*=19)** | ***P*-value** |
| --- | --- | --- | --- |
| **Clinical parameters** | | | |
| Gender, *n* Male/*n* Female | 11/7 | 14/5 | 0.414 |
| Age, years | 29±5 | 28±3 | 0.494 |
| **Laboratory parameters** | | | |
| Log_10_ HBV DNA, IU/ml | 7.4±0.7 | 7.5±0.6 | 0.767 |
| Log_10_ HBsAg, IU/ml | 4.1±0.5 | 4.3±0.5 | 0.143 |
| Log_10_ HBeAg, S/CO | 2.9±0.4 | 3.1±0.1 | 0.095 |
| ALT, U/L | 317±195 | 204±132 | 0.051 |
| HBV genotype, *n* B/*n* C | 14/4 | 13/6 | 0.714 |
