# Supplementary material for: Taurocholic acid inhibits the response to interferon-α therapy in patients with HBeAg-positive chronic hepatitis B by impairing CD8+ T and NK cell function
Source: Cell Mol Immunol. 2021 Jan 11;18(2):461–71. doi: 10.1038/s41423-020-00601-8 (PMC8027018; doi:10.1038/s41423-020-00601-8)
Supplement: Supplementary file 4 — Supplementary Figures 1-7 [file 41423_2020_601_MOESM4_ESM.pdf]

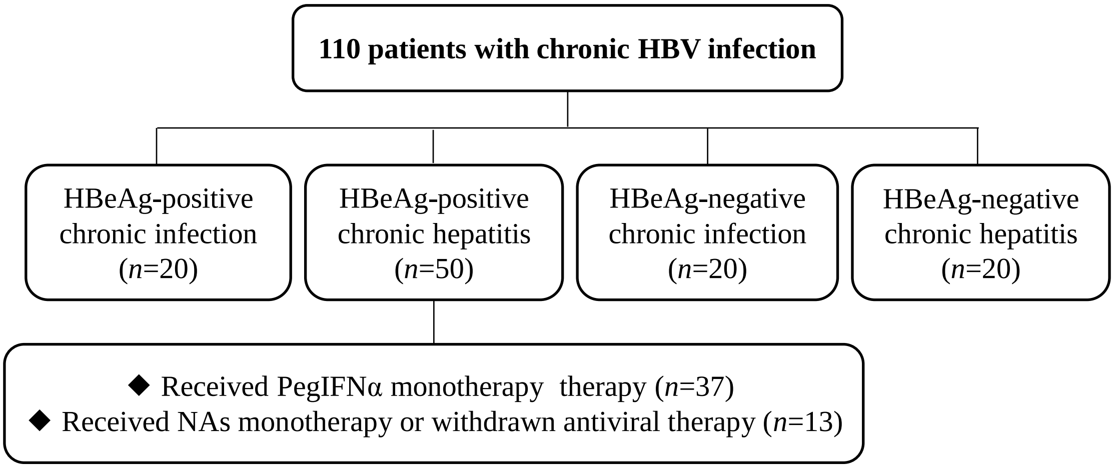

Fig. S1. Flowchart of the patient selection process.

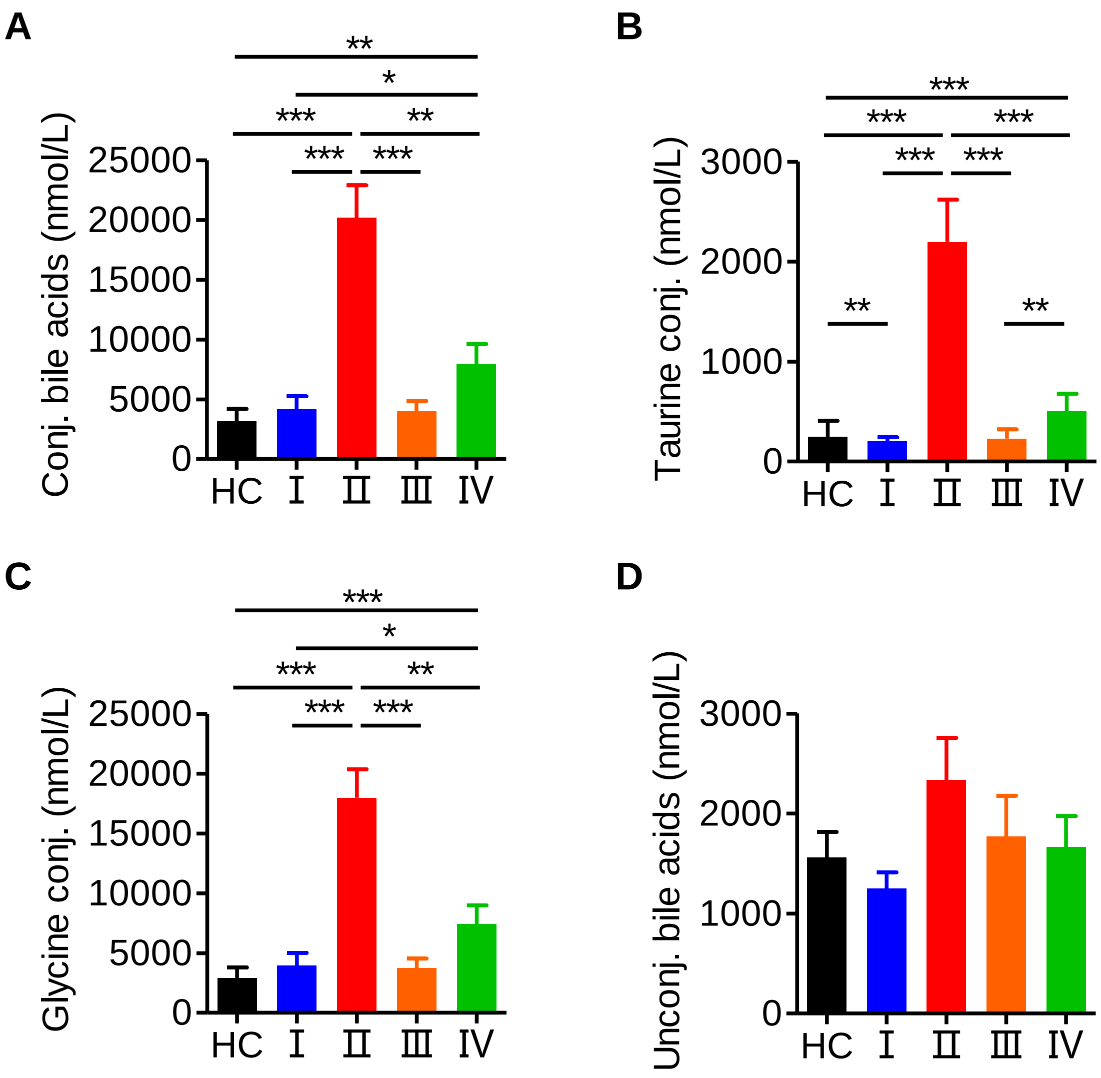

Fig. S2. HBeAg-positive CHB patients have significantly higher serum levels of bile acids than healthy controls and patients in other clinical phases of chronic HBV infection.

Sera of 20 patients with HBeAg-positive chronic HBV infection (I), 50 patients with HBeAg-positive chronic hepatitis B (CHB) (II), 20 patients with HBeAg-negative chronic HBV infection (III), 20 patients with HBeAg-negative chronic hepatitis B (IV) and 20 healthy controls (HC) were analyzed. **(A)** Conjugated bile acids. **(B)** Taurine-conjugated bile acids. **(C)** Glycine-conjugated bile acids. **(D)** Unconjugated bile acids. Data are presented as mean ± SEM. Mann-Whitney *U* test. *$p < 0.05$, **$p < 0.01$, ***$p < 0.001$.

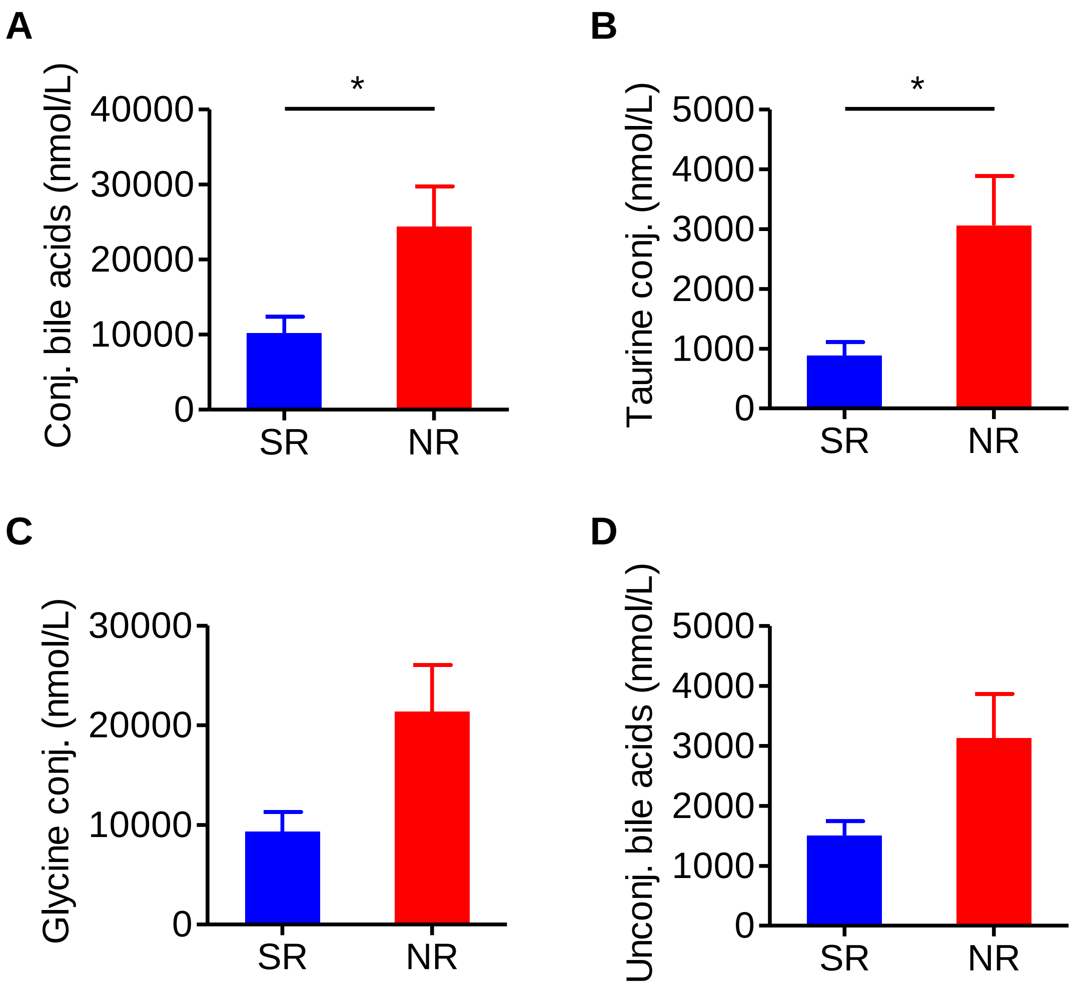

Fig. S3. Non-response patients treated with pegylated interferon-alpha have significantly higher serum levels of bile acids than sustained response patients with HBeAg-positive chronic hepatitis B. Sera of 18 sustained response (SR) and 19 non-response (NR) patients with HBeAg-positive chronic hepatitis B were analyzed. **(A)** Conjugated bile acids. **(B)** Taurine-conjugated bile acids. **(C)** Glycine-conjugated bile acids. **(D)** Unconjugated bile acids.

Data are presented as mean ± SEM. Mann-Whitney *U* test. $^*p < 0.05$.

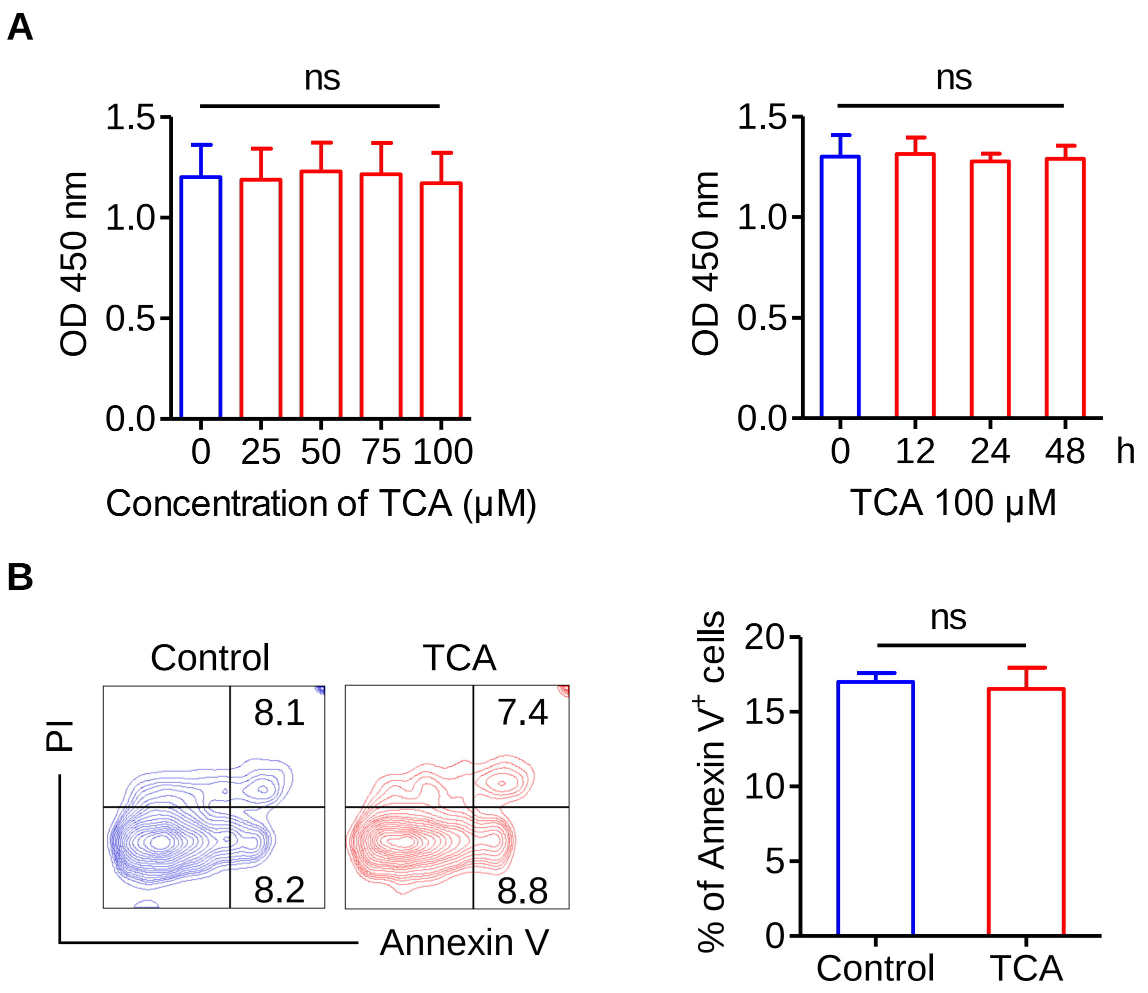

Fig. S4. Taurocholic acid does not induce unspecific immune cell apoptosis *in vitro*.

(A) Cell viability was assessed by CCK-8 assays of freshly isolated PBMCs from HBeAg-positive chronic hepatitis B (CHB) patients stimulated with 100 μM taurocholic acid (TCA) for the indicated times (left) or stimulated with different concentrations of TCA for 24 h (right). **(B)** Freshly isolated PBMCs from HBeAg-positive CHB patients were stimulated with or without 100 μM TCA for 24 h, stained with a combination of annexin V and propidium iodide (PI), and analyzed by flow cytometry. Cells positive for annexin V staining were scored as apoptotic cells.

Data are presented as mean ± SD. Unpaired *t* test. ns, not significant.

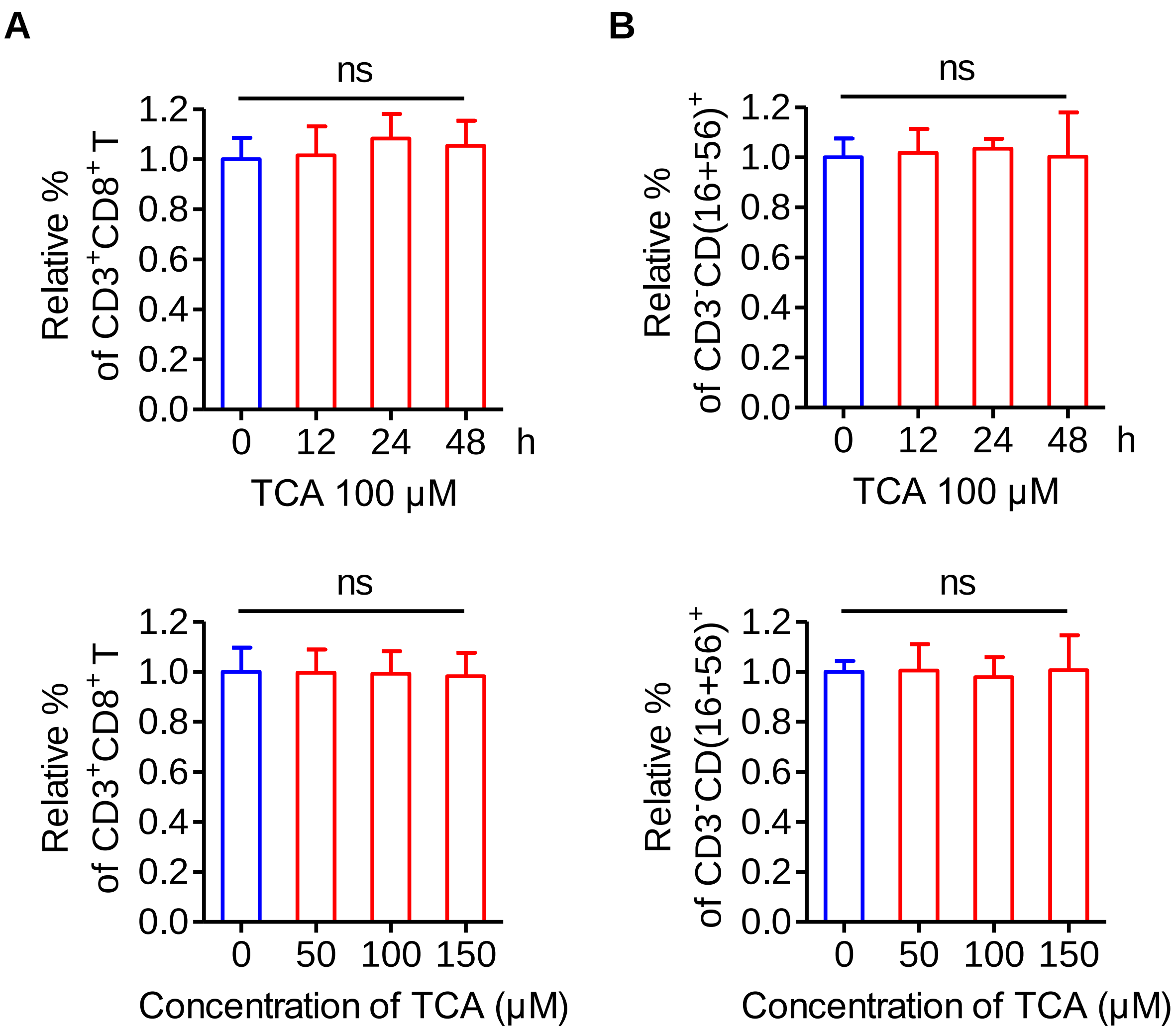

Fig. S5. Taurocholic acid has no effect on the proportion of $CD3^{+}CD8^{+}$ T and NK cells from healthy controls *in vitro*.

The relative proportions of $CD3^{+}CD8^{+}$ T cells **(A)** and NK cells **(B)** were analyzed by flow cytometry of freshly isolated PBMCs from healthy controls stimulated with 100 μM taurocholic acid (TCA) for the indicated times (top) or stimulated with different concentrations of TCA for 24 h (bottom).

Data are presented as mean ± SD. Unpaired *t* test. ns, not significant.

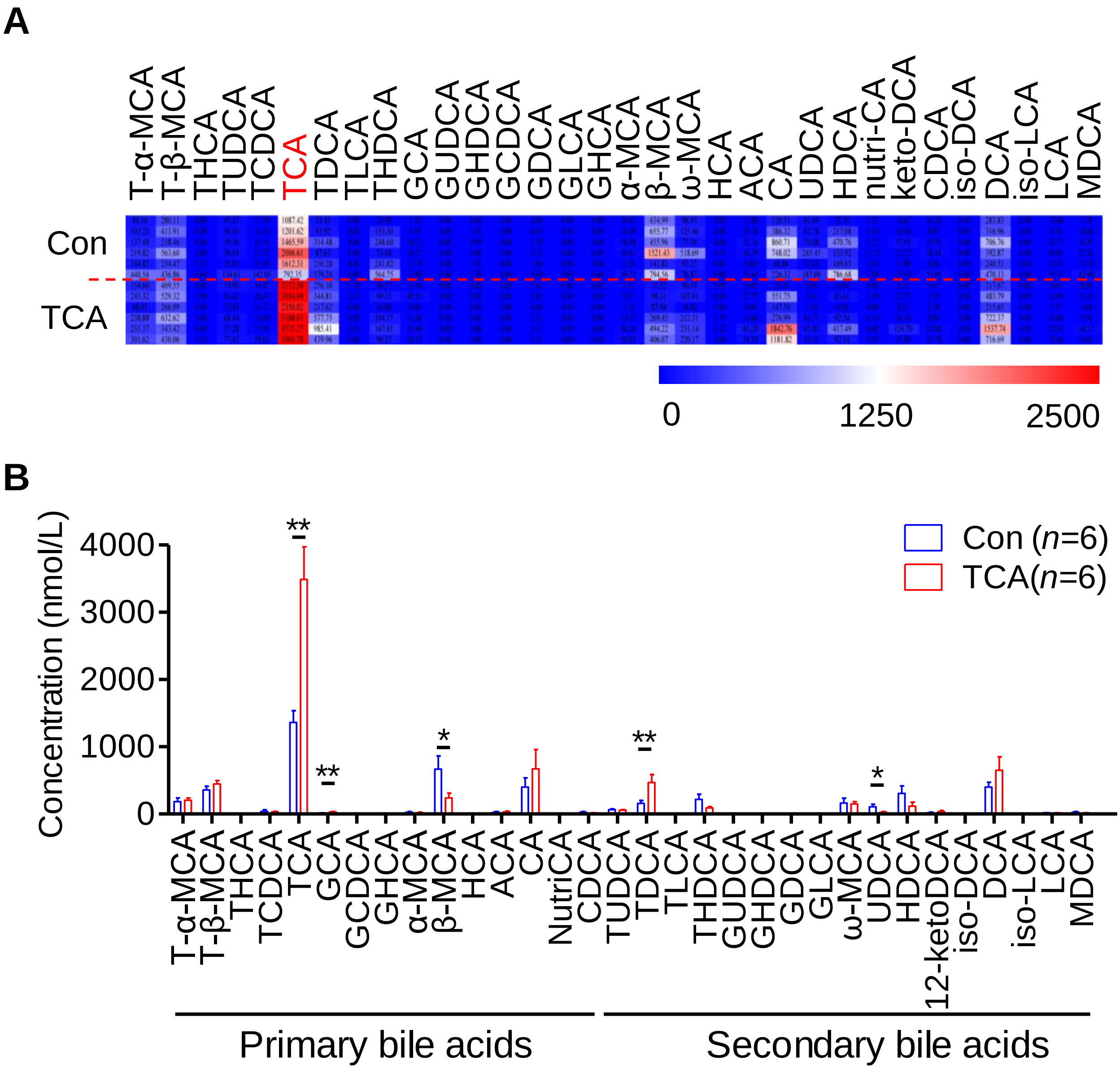

Fig. S6. The serum level of taurocholic acid in C57BL/6 mice is significantly elevated after gavage.

(A) Heatmap of serum bile acid profiles for the control (Con) and taurocholic acid (TCA) groups. **(B)** After 2 weeks of TCA feeding, serum bile acid levels in C57BL/6 mice were measured.

Data are presented as mean ± SEM. Mann-Whitney *U* test. $*p < 0.05$, $**p < 0.01$.

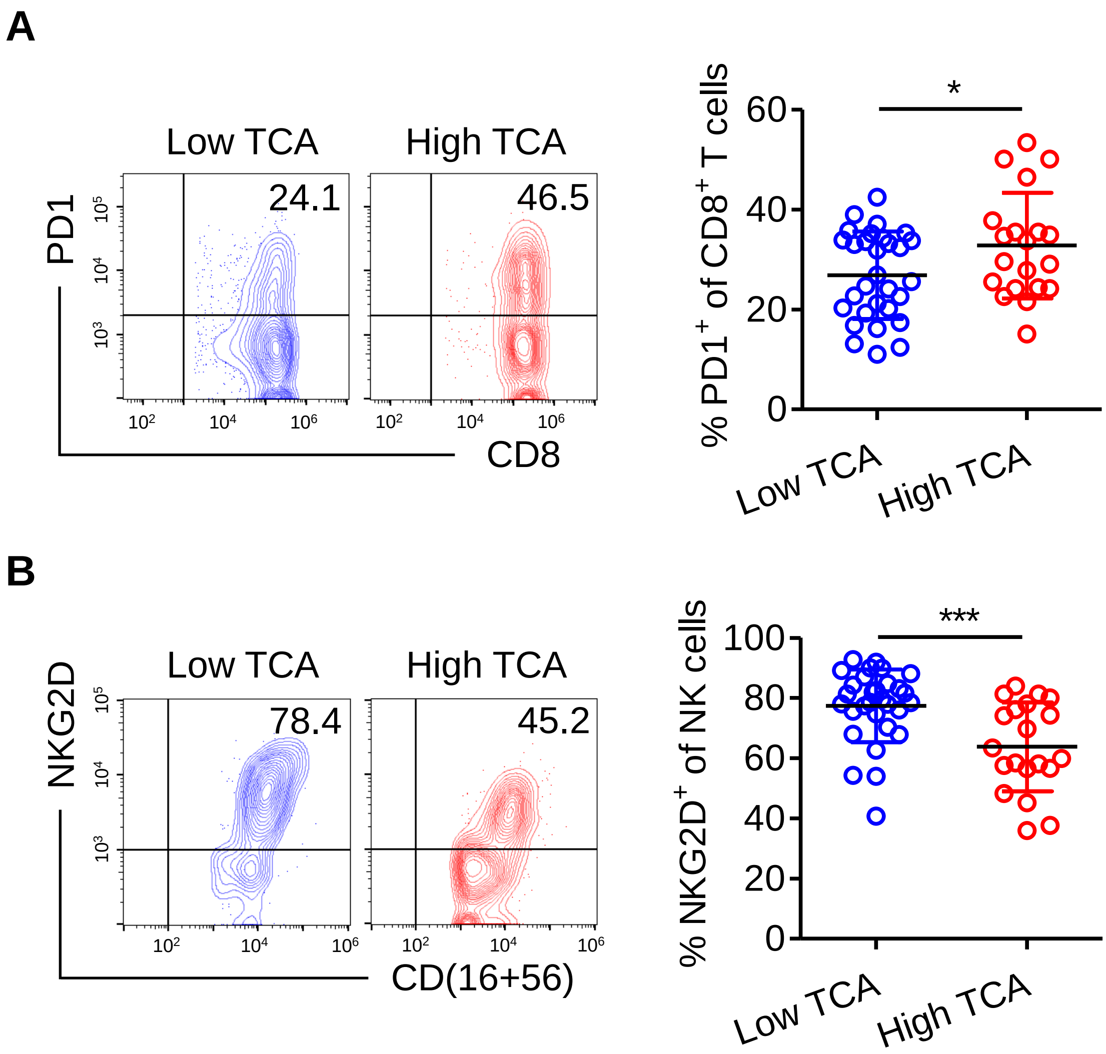

Fig. S7. Taurocholic acid up-regulates PD1 expression on $CD3^+CD8^+$ T cells and down-regulates NKG2D expression on NK cells in HBeAg-positive CHB patients.

Differential PD1 expression on total $CD3^+CD8^+$ T cells **(A)** and NKG2D expression on total NK cells **(B)** within the lymphocyte gate in HBeAg-positive chronic hepatitis B (CHB) patients with low (n=30) or high (n=20) taurocholic acid (TCA) levels.

Data are presented as mean ± SD. Unpaired t test. *$p < 0.05$, ***$p < 0.001$.
